# Effects of Extrusion on Starch Molecular Degradation, Order–Disorder Structural Transition and Digestibility—A Review

**DOI:** 10.3390/foods11162538

**Published:** 2022-08-22

**Authors:** Xiaoyue Huang, Hongsheng Liu, Yue Ma, Shihua Mai, Cheng Li

**Affiliations:** 1 School of Food Science and Engineering, South China University of Technology, Guangzhou 510641, China; 2 School of Health Science and Engineering, University of Shanghai for Science and Technology, Shanghai 200093, China

**Keywords:** extrusion, starch structure, starch digestibility

## Abstract

Extrusion is a thermomechanical technology that has been widely used in the production of various starch-based foods and can transform raw materials into edible products with unique nutritional characteristics. Starch digestibility is a crucial nutritional factor that can largely determine the human postprandial glycemic response, and frequent consumption of foods with rapid starch digestibility is related to the occurrence of type 2 diabetes. The extrusion process involves starch degradation and order–disorder structural transition, which could result in large variance in starch digestibility in these foods depending on the raw material properties and processing conditions. It provides opportunities to modify starch digestibility by selecting a desirable combination of raw food materials and extrusion settings. This review firstly introduces the application of extrusion techniques in starch-based food production, while, more importantly, it discusses the effects of extrusion on the alteration of starch structures and consequentially starch digestibility in various foods. This review contains important information to generate a new generation of foods with slow starch digestibility by the extrusion technique.

## 1. Introduction

As the main source of carbohydrates and energy in the diet, starch has a critical role for human health. Starch in food can be divided into rapidly digestible starch (RDS), slowly digestible starch (SDS) and resistant starch (RS) according to its digestion rate and location in the human gastrointestinal tract [1]. Consumption of RDS could lead to a rapid increase in postprandial blood glucose levels, which can further induce chronic metabolic diseases such as obesity, type 2 diabetes and cardiovascular disease [2]. SDS is slowly digested and absorbed in the small intestine, which is helpful in terms of maintaining a stable postprandial blood glucose and insulin response [3]. RS cannot be digested in the small intestine, while it can be partially or completely fermented in the colon into short-chain fatty acids, which play an important role in improving gut health [4]. The health benefits of SDS and RS have encouraged both academia and industry to develop starch-based foods with either slowly digestible or resistant starch properties.

Extrusion has been widely applied in the production of starch-based foods, such as biscuits, pasta and breakfast cereals, due to its convenience, high production efficiency and low cost [5]. During extrusion, food macronutrients including starch undergo order–disorder structural transitions, such as starch gelatinization, degradation (breakage of starch molecules) and retrogradation (e.g., the formation of amylopectin double helices and amylose–lipid complexes) [5]. These starch structural alterations during extrusion are largely related to the final starch digestibility in food products. Native starch granules are inherently more difficult to digest compared to gelatinized starch, because of its natural crystalline and granular structures [6]. The digestion rate of gelatinized starch can be significantly reduced after retrogradation, which promotes the formation of some short-range and long-range ordered crystalline structures [7]. Therefore, depending on different sources of starch raw materials and their distinct gelatinization/retrogradation properties, large variance in starch digestibility could be created in the extruded foods [8]. Small variations in extrusion processing conditions can also cause a significant difference in starch structural ordering state after extrusion and consequently result in the distinct starch digestibility of extruded foods [9]. Therefore, it is conceivable that by selecting a desirable combination of starch raw materials and extrusion processing conditions, extruded food products can be developed with slow starch digestibility.

This review aims to firstly introduce the extrusion technique and its application in the food industry, followed by a comprehensive review on the effects of extrusion on starch structures and digestibility, as well as corresponding mechanisms. Although some reviews have discussed the effect of extrusion on starch digestibility, this paper attempts to explain the digestibility of extruded starch from the perspective of different starch structure levels. This review could thus act as the basis for the development of extruded starch-based foods with slow starch digestibility (i.e., low glycemic index) in the future.

## 2. Brief Introduction of Extrusion Technique

The aim of this section is to briefly introduce the extrusion technique, which could serve as the basis to better discuss and understand its effects on starch structures and digestibility in following sections. For further details on the extrusion technique, readers are encouraged to refer to other previous reviews [10].

**Figure 1:**
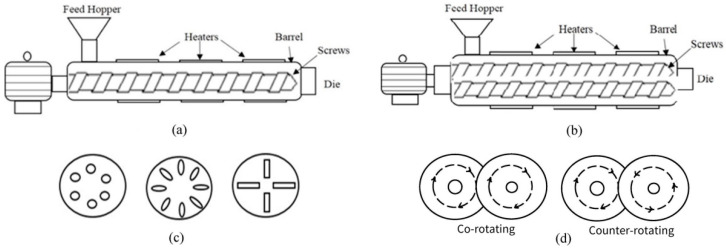
Types of food extruders, including (**a**) single-screw extruder, (**b**) twin-screw extruder, (**c**) types of die openings and (**d**) co- and counter-rotating screw. Reprinted with permission from Ref. [12]. 2022, Elsevier.

According to the number of screws, extruders can be divided into single-screw, twin-screw and multi-screw extruders (Figure 1) [11]. There is only one screw in the barrel of single-screw extruders, which can transport feed materials by the friction between screw and barrel. The shortcoming of single-screw extruders is that they can easily be blocked and leak and are only suitable for simple cooking and molding. According to the rotation direction and assembly mode of screws, twin-screw extruders can be further divided into co-rotating and counter-rotating, and meshing and non-meshing extruders. They rely on the engagement of two screws to convey materials forcibly, so feed materials do not easily flow backwards. It also has a unique self-cleaning function and can adapt to more complicated processing needs [11]. Therefore, twin-screw extruders are more widely used compared to the single-screw extruders nowadays. Multi-screw extruders are rarely used in food processing because of their difficult manufacturing [11].

The extrusion process can be generally described as follows: raw material enters the extruder from the feeding cavity and is transported forward in the barrel under the force of the rotating screw. Due to the friction among the screw, material and inner wall of the barrel, the material is strongly squeezed, stirred and sheared, resulting in further refinement and homogenization of the material. With the continuous increase in pressure and temperature in the barrel cavity, the raw material could be melted and changed from a solid state to fluid state when certain moisture content is present, accompanied by a series of physical and chemical reactions. When the material is finally discharged from the die of the extruder, the water from the extruded material evaporates quickly and produces a huge expansion force owing to the instantaneous pressure drop, which causes the extruded material to instantly expand, thereby forming a loose, porous and crispy expanded product [11,13]. Many physical, chemical and textural changes take place in the extrusion process, such as starch gelatinization and degradation [14]. Readers are encouraged to refer to other comprehensive reviews to gain a better understanding of the effects of extrusion on structural and property alterations in different food macronutrients [15,16].

There are many advantages of applying extrusion technology in food production. For example, the die of the extruder can be designed into a variety of complex structures (Figure 1c), thus giving the extruded food various shapes, such as rod, ball, tube, curved cylinder and cone. After molding, the extrudate is cut into a suitable length by a blade rotating along the surface of the die. In the extrusion process, functional ingredients or fortified nutrients such as vitamins and minerals can be added to improve the nutritional value of different foods and meet the dietary nutritional needs of special individual groups, such as infants and the elderly. In addition, extrusion can be used as a pretreatment method to improve the processing characteristics of raw materials or the functional characteristics of food ingredients [17]. For example, extrusion can improve the hydration and gelatinization properties of natural flour and increase its application potential in different food products [18]. The pregelatinized starch prepared by improved extrusion cooking technology (IECT) showed improved gel stability and a lower short-term retrogradation rate [19]. Shorter amylopectin fragments with fewer intermolecular associations formed by extrusion processing have the effect of retarding the staling of bread and cakes [20].

## 3. Effects of Extrusion on Starch Structures

Starch has a complex structure, which can be generally divided into five levels [21]. Individual starch chains are the first-level structure, which are further connected through α-(1,6) glycosidic linkages into amylose or amylopectin molecules (second-level structure). Amylopectin side chains (especially A and B1 chains) can form double helices into clusters (third-level structure), with the branching points located in the amorphous area [22]. These semi-crystalline lamellae are alternated with amorphous growth rings (fourth-level structure) into starch granules (fifth-level structure). The first- to second-level structures are frequently referred to as the starch molecular structure, and the third- to fourth-level structures can be generally referred to as the crystalline structure.

Each level of starch structure can potentially have a significant effect on the final starch digestibility in different food products, depending on their order–disorder structural transition during food processing, as well as their physical, chemical or combined modifications. The major difference in starch digestibility in various foods is related to whether the starch is ingested native, gelatinized or retrograded. Native starch is inherently difficult to digest as it has crystalline (such as long-range ordered structure (crystals) and short-range ordered structure (double helices)) and granule structures (such as the size, shape and surface morphology of starch granules) [6]. Crystalline and granule structures can thus largely determine the starch digestibility at its native state [23]. For example, native potato starch has a slower digestion rate than maize starch due to the lack of granule surface pores [23]. Starch granules heated in the presence of water undergo an order–disorder phase transition called gelatinization, which commonly involves water uptake, granular swelling, the unwinding of double helices, loss of birefringence, starch solubilization and viscosity development. After complete gelatinization, all starch crystalline structures (such as hexagonal and orthorhombic crystals) would be destroyed, which can substantially promote starch digestibility [24]. Dispersed starch molecules after gelatinization could re-associate into crystallites through both inter- and intramolecular interactions during the cooling period [25], a process known as starch retrogradation. Hexagonal crystallites are frequently formed with the progression of retrogradation [26]. Although the crystallites or entanglements formed during retrogradation are less stable than those from the native starch, these crystallites are still able to largely inhibit or restrict their binding affinity and catalysis activity towards starch digestive enzymes due to their bulk configuration, which can subsequently result in a slower starch digestion rate and higher RS content [27]. For instance, the amount of RS and SDS is significantly increased after starch retrogradation on cooling [28], possibly due to the formation of perfectly and imperfectly packed crystallites during retrogradation, respectively. Starch molecular structures are thus the driving factors in determining the starch digestibility in both gelatinized and retrograded states, as the higher levels of starch structures, such as amylopectin double helices, have been destroyed during gelatinization [29].

Therefore, it is conceivable that by modifying the order–disorder state of starch, including the long-range ordered structure (crystals) and short-range ordered structure (double helices), in various food products by extrusion could largely determine their starch digestibility. The following section is thus focused on the effects of extrusion on different levels of starch structures, aiming to elucidate the mechanisms by which extrusion affects starch structures and digestibility.

### 3.1. Starch Molecular Structure

Starch is a glucose polymer, which can be divided into amylose and amylopectin. Amylose typically accounts for 20–30% of starch and is an essentially linear molecule linked with α-1,4-glycosidic bonds. Amylopectin is a highly branched molecule connected by α-1,4 and α-1,6-glycosidic bonds. Starch molecules could undergo significant degradation during extrusion, which has a critical effect on the physiochemical properties and digestibility of starch [30]. Therefore, a comprehensive characterization of starch degradation degree during extrusion is crucial in terms of understanding the extrusion processing–starch structure–starch property relations. Currently, starch molecular structures are frequently characterized by the chain-length distribution (CLD) and molecular size distribution of amylose and amylopectin molecules [31]. Different techniques, including size-exclusion chromatography (SEC), fluorophore-assisted capillary electrophoresis (FACE), high-performance anion-exchange chromatography (HPAEC) and asymmetric flow field fractionation (AF4), have been applied to obtain the starch CLD and molecular size distribution [32]. The advantages and limitations of each technique in terms of characterizing the starch fine molecular structure can be found in earlier reviews [33].

**Figure 2:**
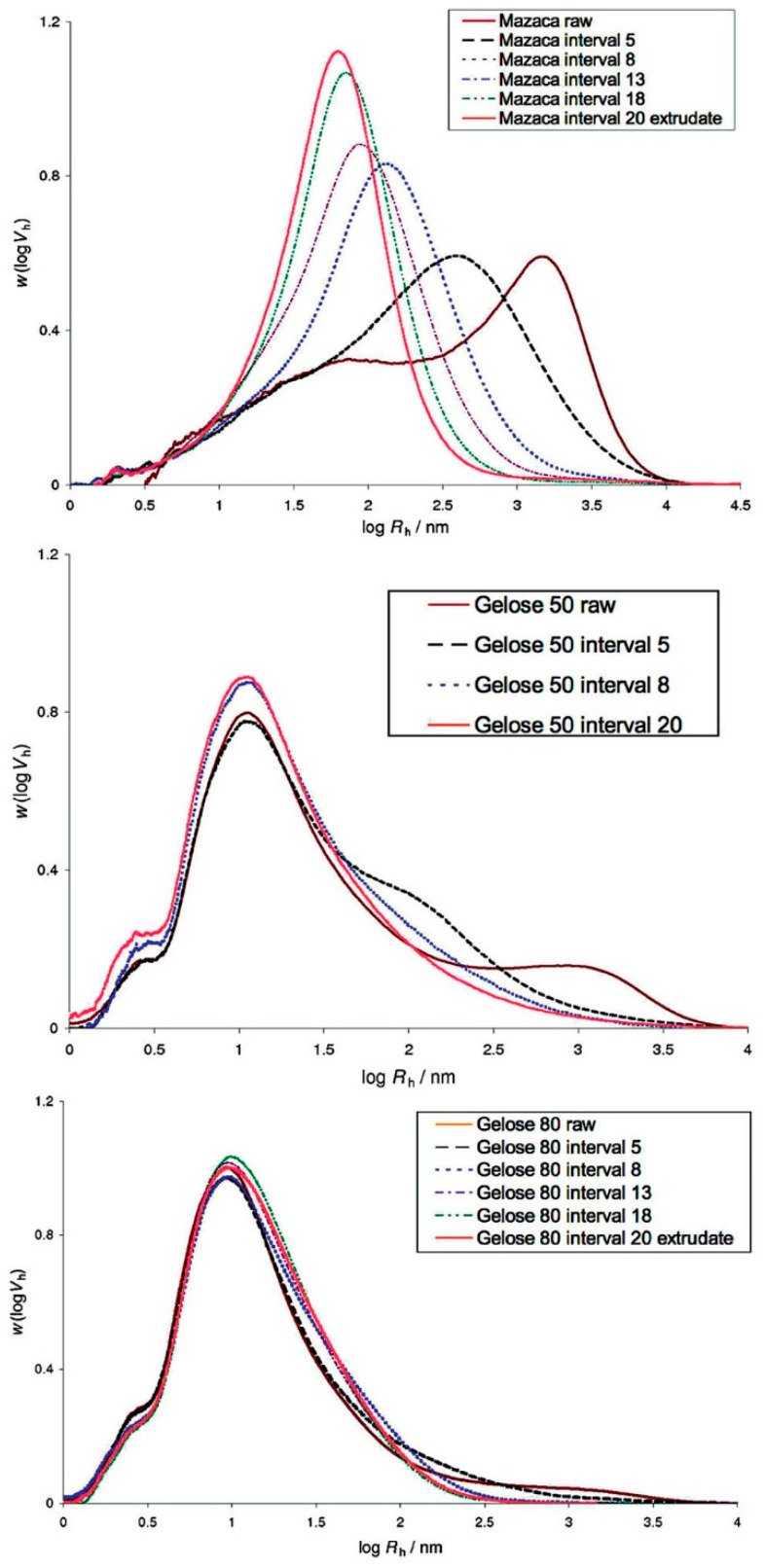
Molecular size distribution evolution of Mazaca (**upper**), Gelose 50 (**middle**) and Gelose 80 (**bottom**) starch during extrusion. The barrel was divided into 20 intervals along the conveying direction for sample collection purposes. Starch samples were collected at different barrel intervals (e.g., 5, 8, 13, 18 and 20) after the torque of the extruder reached a steady state. Reprinted with permission from Ref. [35]. 2022, ACS Publications.

The high temperature, pressure and mechanical shearing force in the extrusion process can easily break hydrogen bonds among starch molecules, especially with the amylopectin molecules [34]. As shown in Figure 2, the size distribution of fully branched Mazaca starch (0% amylose) is wide and left-skewed at the beginning of extrusion, while the amylopectin peaks of Gelose 50 corn starch (55% amylose) and Gelose 80 corn starch (85% amylose) are much smaller [35]. As extrusion proceeds, amylopectin molecules are continuously degraded and converge towards the maximum stable size, while there is a much less significant change in the size distribution of amylose molecules [35]. Finally, the degraded amylopectin and amylose molecules reveal a single size distribution peak. It seems that amylopectin is more susceptible to shear degradation than amylose, possibly due to its large molecular size and highly branched structure [35]. As a result, the proportion of molecules in the molecular size range of amylose increases after extrusion due to the significant degradation of amylopectin molecules into smaller molecules [36]. Table 1 shows the changes in starch molecules and amylose content during extrusion. Many studies have found that the amylose content of starch does not significantly change, regardless of extrusion processing conditions [37,38,39]. Sarawong et al. [40] reported that the amylose content of green banana flour increased significantly due to the cleavage of α-1,6 glycosidic bonds of amylopectin under shearing force in the extruder. In contrast, the extruded high-amylose corn flour had decreased amylose content because of the leaching of amylose and the formation of complexes with lipids [41].

On the other hand, SEC CLD results show that amylose also undergoes degradation during extrusion, causing the production of shorter amylose chains and the loss of iodine binding capacity by these amylose chains [42]. Interestingly, amylopectin CLD does not change significantly after mild extrusion, suggesting that degradation mainly occurs near the branching points [19,39]. Meanwhile, extensive extrusion could further degrade the α-(1, 4) glycosidic bonds in amylopectin chains [38]. Based on the results of Brummer et al. [43], the significant cleavage of α-1,4 linkages would only occur when the product temperatures exceed 180 °C. Mechanical shear is believed to be the main mechanism of starch molecular degradation during extrusion [14,44]. On the other hand, heat energy can gelatinize starch and reduce its viscosity; thus, it may indirectly aid in the degradation of starch molecules [36].

As mentioned above, the degradation of starch molecules is of significance in terms of determining the starch digestibility in both its gelatinized and retrograded state. Excessive shearing may cause amylose to degrade into molecules with a much shorter chain, which cannot retrograde efficiently, inhibiting the formation of retrograded amylose double helices, which are known as type 3 RS (RS3) [45]. Small dextrin degraded from amylopectin may also inhibit the aggregation of amylose during retrogradation [39]. On the other hand, smaller amylopectin molecules have a faster retrogradation rate through both inter- and intramolecular interactions compared to the undegraded amylopectin; retrograded amylopectin double helices mainly contribute to the formation of SDS instead of RS [46]. Therefore, by optimizing the starch molecular structure through extrusion, one could potentially slow down the starch digestibility in extruded food products.

### 3.2. Starch Crystalline Structure

Starch granules are composed of semi-crystalline and amorphous growth rings (approximately 100~400 nm thickness), which can be frequently characterized by X-ray diffractometry (XRD) and small-angle X-ray scattering (SAXS) techniques [47]. The semi-crystalline growth ring is further composed of alternating amorphous and crystalline lamellae with a periodicity of 9–10 nm [48,49]. The crystalline lamellae are formed by ordered amylopectin double helices, whereas the amorphous lamellae consist of amylose chains and amylopectin branching points [32,50]. According to the different X-ray diffraction characteristics, natural starch can be divided into A, B and C types, among which the C-type diffraction pattern is a mixture of the A and B types [51,52]. A-type starches (e.g., wheat, rice and corn) normally have orthorhombic crystalline structures, B-type starches (e.g., high-amylose starches) contain hexagonal crystalline structures, and the X-ray diffraction patterns of C-type starches (e.g., legume, root, some fruit and stem starches) exhibit simultaneously both the orthorhombic and hexagonal crystalline structures [53,54]. In addition, it is proposed that amylose can form a V-type crystallinity polymorph with both endogenous and extrinsic lipids, where the amylose chain is present as a left-handed single helix [55]. However, this crystal structure has not been identified in crystallography. Under a polarized microscope, starch granules give rise to birefringence (commonly referred to as Maltese cross), which is a characteristic of the crystalline structure, indicating the radial anisotropy of the molecular chain arrangement within starch granules [56].

As mechanically explained in the second section, extrusion involves both thermal and mechanical energy, which could largely damage the starch crystalline structure, including the long-range ordered structure (crystals) and short-range ordered structure (double helices). During extrusion, starch granules could rapidly absorb water and swell, leading to starch gelatinization. On the other hand, after extrusion, melted starch molecules could be re-associated into double helices during cooling, leading to starch retrogradation. Therefore, starch undergoes a transition between order and disorder structures (as mentioned above) over the extrusion process, involving both starch gelatinization and retrogradation; this process could significantly determine the starch digestibility in different starch-based foods [57].

**Figure 3:**
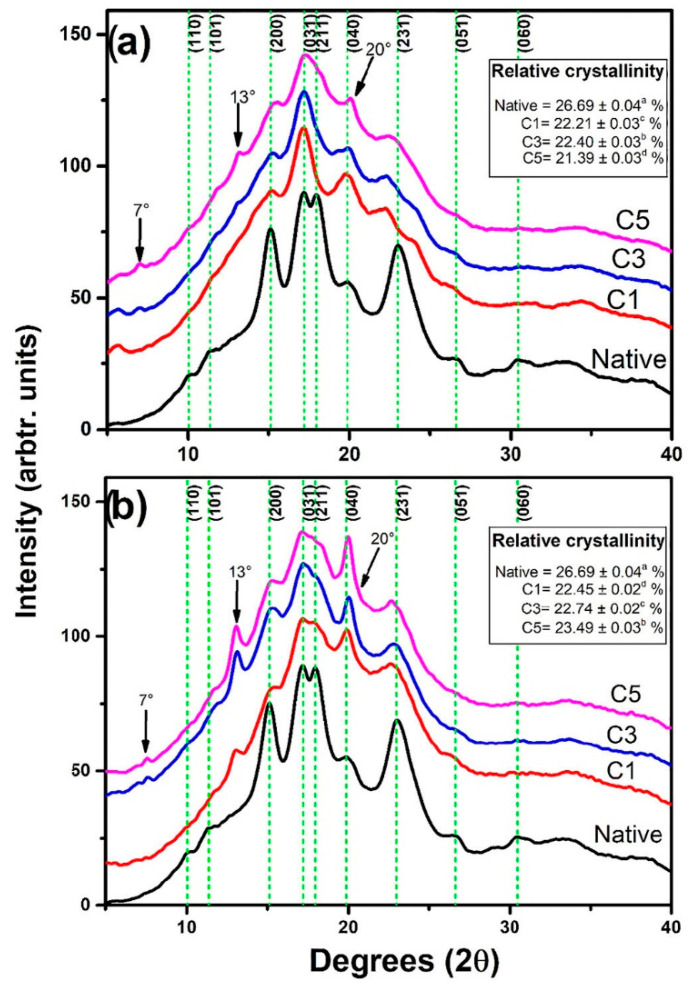
X-ray diffraction patterns of native corn starch and corn starch subjected to heating–cooling extrusion cycles at different temperatures: (**a**) 100 °C and (**b**) 125 °C. C1: one extrusion cycle. C3: three extrusion cycles. C5: five extrusion cycles. Reprinted with permission from Ref. [58]. 2022, Elsevier.

According to XRD results, the original diffraction peak intensity of native starch obviously decreases or disappears, and there may be new diffraction peaks appearing after extrusion (Figure 3) [58]. The new peaks appearing at 2θ of 7°, 13° and 20° are related to V-type crystals and explained as the result of the lamellar growth of starch crystals after the breakage of orthorhombic crystals [58]. Biliaderis [55] showed that extrusion at high temperatures (>185 °C) can form a new crystalline structure called the Eh type, characterized by a different interaxial helical distance and slight shift in the diffraction peak to a lower diffraction angle compared to the Vh-type crystallinity structure. Similarly, von Borries-Medrano et al. [59] reported that the extruded sample showed a peak (Eh pattern) at 2θ of 18.4°, although this crystal structure has not been indexed. In their view, due to the low degree of chain arrangement, the Eh-type crystallinity structure is unstable and would irreversibly transform into the Vh crystallinity structure when the moisture content is increased [55]. However, from a crystallographic point of view, there is currently no evidence for the formation of these two crystalline structures. The effects of extrusion on starch crystalline structure also depend on the starch molecular structures. For example, high-amylose starch could still fully or partially retain its crystallinity structure after extrusion, compared to the normal or waxy starch, which could be related to its much higher gelatinization temperatures [60].

As mentioned above, the digestion rate of native starch is largely determined by the starch crystalline structure. For example, hexagonal crystals are more difficult to be digested by starch digestive enzymes than orthorhombic crystals [61]. Amylose–lipid complexes are known as type 5 RS (RS5) [62]. Therefore, extrusion could potentially modify starch digestibility at its native state through the alteration of the starch crystalline structure. Furthermore, starch gelatinization increases the availability of starch towards digestive enzymes, which causes the substrate to be more easily digested [63]. Starch digestibility is therefore often positively correlated with the degree of gelatinization [24]. The starch gelatinization degree can thus be modified by controlling the extrusion raw materials, temperature, moisture, screw speed, die size, feed rate and other factors in order to achieve desirable starch digestibility in extruded foods [64]. For example, different degrees of gelatinization of high-amylose maize starch were developed by extrusion, which resulted in a significant difference in the starch digestibility [65]. Similarly, it has been found that the RDS content was significantly increased while RS content decreased after the extrusion of soybean flour, possibly related to starch gelatinization [8]. On the other hand, as mentioned in the above section, extrusion could reduce the amylopectin molecules’ size, which can promote the retrogradation of amylopectin molecules and formation of SDS due to the formation of unstable double helices [66]. This was supported by the addition of extruded banana starch to bread, which resulted in a significant increase in SDS content [63].

### 3.3. Starch Granule Morphology

There is large variance in starch granule structures among different starch sources due to the influence of genetics and the growing environment, which can be frequently characterized by scanning electron microscopy (SEM). For example, the size of starch granules varies from less than 1μm to more than 100 μm in diameter [67]. Potato starch granules (15~100 μm) are larger than those of cereal starch, such as rice starch granules (3~8 μm) [68]. Wheat starch granules have a bimodal size distribution, with a mean diameter of 15~40 μm and 1~10 μm [61]. Starch granules can have round, spherical, oval, lenticular, polygonal or irregular shapes. For example, potato has an oval starch granule, and rice has a polyhedral starch granule [69]. Another important structural feature of starch granules is the presence of pores, channels and cavities [70]. For example, corn, sorghum and millet starch have surface pores [71]. These pores connect the surface to the helium of starch granules [72,73], which allows the entrance of starch digestive enzymes [74]. Therefore, starches with surface pores are frequently digested at a faster rate than those without surface pores [71].

**Figure 4:**
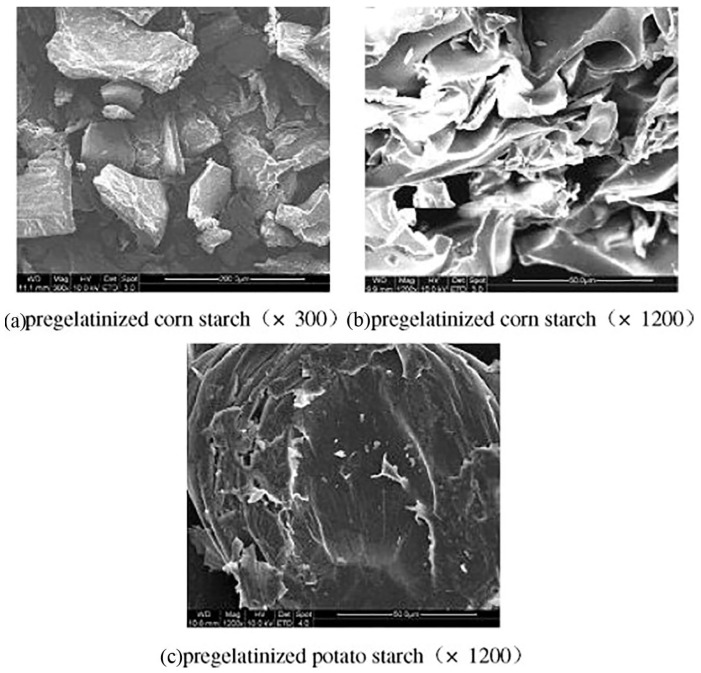
SEM microstructures of pregelatinized starch granules by extrusion. Reprinted with permission from Ref. [75]. 2022, Elsevier.

Extrusion could largely destroy the structural integrity of starch granules through fragmentation and finally results in irregular hole-shaped structures due to the sudden drop in pressure and evaporation of water when starch material is extruded from the die (Figure 4) [30,75]. It has been found that the pore wall becomes thicker, the pore size is enlarged, while the number of pores decreases, over increasing moisture content and feeding rate during extrusion [76]. However, it has been reported that some starch crystallites could still remain after extrusion [60]. Similarly, it has been found that starch molecules inside the granule fragments remain intact, while those on the surfaces of the fragments are more likely to be shear-degraded [44].

Extrusion destroys the compact granular structure of starch and turns it into a loose and porous structure, which may promote the starch digestion rate and lead to less starch reaching the colon (a decrease in the RS content) [77]. Furthermore, the starch granule surface structure can also significantly affect the starch digestibility. For example, rougher surfaces (with pores and cracks) can supply more binding sites for starch digestive enzymes and thus cause a faster digestion rate than a starch material with a smoother surface (such as potato starch) [78]. Therefore, extrusion could also have a significant effect on the starch digestibility by altering the starch granule surface structure.

## 4. Effects of Extrusion Parameters on Starch Digestibility and Possible Mechanisms

### 4.1. Raw Material Composition

Raw material characteristics such as the source and size of starch granules, amylose/amylopectin ratio, lipid, protein and dietary fiber content could largely determine the digestibility of extruded starch. Bean genotype has been proven to affect the digestibility of starch, largely due to the varied organization and amylose content of starch granules [8]. Depending on the botanical source, the crystal types and crystallinity of starch vary. The RS content of extruded kidney bean (16.3%) and field pea starch (15.6%) is much higher than that of extruded corn starch (2.5%) [79]. This could be attributed to the C-type crystalline structure of native kidney bean and field pea starch (containing a mixture of both orthorhombic and hexagonal crystals) compared to corn starch, which has an orthorhombic crystal structure. Hexagonal crystals have more branch points clustered in the amorphous region, which are more rigid and resistant to enzymatic hydrolysis than orthorhombic crystals. Particle size is another critical factor in determining the starch digestibility after extrusion. For example, finely milled sorghum and barley grains have higher digestibility than medium and coarsely milled grains after extrusion, due to their higher specific surface area and therefore enlarged area for contact with starch digestive enzymes [80]. The particle size can also affect the rate and extent of water penetration during starch gelatinization, thus further affecting starch digestibility [80]. The formation of RS during the extrusion process is mainly attributed to the retrogradation of amylose molecules [45]. As a result, amylose content is a critical factor in determining starch digestibility after extrusion, and high-amylose starches generally form more RS than normal or waxy starches [41,81]. Lipid content could act as a lubricant during the extrusion process, which reduces the mechanical force and degradation of starch macromolecules, further affecting the starch digestibility [82].

Amylose content and structure (e.g., chain-length distribution) can affect the extruded starch digestibility by forming complexes with both endogenous and extrinsically added lipids, which is known as RS5 [16]. This can be explained by the decrease in amylose solubility and conformational hindrance of V-type crystals to enzyme attack [83]. The formation of amylose–lipid complexes has been found in many studies during the extrusion process, and the amount of amylose–lipid complexes depends on the type of both starch and lipid components present in the food [58,84]. Monoglycerides and free fatty acids more easily form complexes than triglycerides [84], as triglycerides are too large to enter into starch spirals to form stable helices. Within a certain range, the higher the amylose content, the shorter the fatty acid chain length, and the more complexes could be formed [85]. The ability of amylopectin to form complexes with fatty acids is much weaker than that of amylose, mainly due to the short side chain and steric hindrance of amylopectin [86]. Saturated fatty acids are favored over unsaturated ones in amylose–lipid complex formation since the molecular rigidity of the double bonds in unsaturated fatty acids hinders access to the amylose helix [87].

Protein can form a matrix around starch granules, delaying or preventing its contact with digestive enzymes, thereby reducing starch digestibility [88]. For example, beans frequently have a slow starch digestion rate due to their high protein content [89]. During the extrusion process, protein can also restrict the swelling and gelatinization of starch granules, thus reducing the starch digestibility [90]. Dietary fiber in raw feed materials could also affect starch digestibility. For instance, the addition of tomato and grape pomace could reduce the starch digestibility of barley extrudates, possibly due to the fact that starch granules are trapped in the protein–fiber–starch network structure [91]. Apple pomace could limit starch gelatinization by reducing the starch hydration level, which reduces the total digestible starch content [92].

### 4.2. Feed Moisture, Extrusion Temperature and Screw Speed

Feed moisture has a significant effect on both starch structure and digestibility during extrusion (Table 2). As the moisture content increases, the lubricating effect is enhanced, reducing the friction among raw material, screw and barrel [12]. High moisture content could further reduce the viscosity and shorten the residence time of starch material in the extruder cavity, thus weakening the shearing effects on starch molecules [93].

In terms of starch digestibility, the influence of moisture content depends on the balance between starch gelatinization and retrogradation (Table 2) [94]. Starch gelatinization mainly occurs at high moisture content during extrusion [95], which might increase the susceptibility of starch towards digestive enzymes. On the other hand, it is believed that starch retrogradation is enhanced at relatively high moisture content during extrusion, which could promote the formation of more RS3 [96]. This is partially due to the fact that water acts as a plasticizer to decrease the starch glass transition temperature so that starch molecules are more flexible to rearrange into double helices during retrogradation [96,97]. For example, the extrusion of a sorghum–barley mixture at moisture above 30% produced more RS [98]. Similarly, Kim et al. [99] found that higher feed moisture content and a longer storage time can significantly increase the RS content (by~11 times). The RDS content of extruded brown rice and pinto bean flour mixture was decreased while RS increased with the increase in processing moisture [100]. However, contradictory results have also been found in the literature. For example, it has been found that higher RS content was produced by extrusion under limited moisture content, which was rationalized by the notion that extrusion could produce a higher amount of smaller molecules and shorter chains due to the lack of lubrication of water under limited moisture content, and these degraded starch molecules have higher mobility during cooling, promoting the formation of RS [101]. A summary of the effects of different extrusion parameters on starch digestibility is shown in Table 2.

Higher temperatures can frequently promote starch gelatinization and the destruction of starch crystallites during extrusion, which can thus increase the starch digestibility [108]. For example, corn and potato starches extruded at higher temperatures revealed higher in vitro digestibility than those extruded at lower temperatures [105]. In addition, the extrusion temperature can affect the formation of amylose and lipid complexes. It is believed that there is an optimal temperature for lipids with different fatty acid chain lengths to form complexes with amylose molecules [109,110]. Gulzar, Hussain, Naseer and Naik [103] found that with the increase in barrel temperature, a crystalline structure that is more stable to heat and decomposition is formed, showing an increase in RS content.

Screw speed is another critical factor affecting starch digestibility (Table 2). With the increase in screw speed, the friction and shearing force on raw starch-based material increase, which further increase the extent of starch destruction and degradation [96,111]. However, if the screw speed is too high, the residence time of feed material in the barrel is largely shortened, resulting only in insufficient starch gelatinization [112]. Therefore, the effect of screw speed on starch digestibility is determined by the combination of shearing action and residence time. For example, for sorghum extrudates, maximum starch digestion was produced at a moderate moisture level (30%) and screw speed (250 rpm) [94]. Conversely, Brahma, Weier and Rose [101] found that moderate screw speeds (300 rpm) tended to enhance SDS and diminish RDS, because either an overly long residence time or an overly high shear rate was not conducive to preserving the slowly digestible property of starch. In addition, it was reported that the starch digestibility of sorghum–barley extrudates was not significantly affected by the screw speed [98]. The effects of screw speed on starch digestibility are also related to other conditions, such as raw material properties and screw configuration, so the conclusions of different studies have contradictory trends.

Besides moisture content, extrusion temperature and screw speed, other important extrusion variables, such as feed rate, screw configuration and die design, will also affect starch digestibility. The feed rate mainly affects the residence time of the material in the extruder barrel, which further affects the degree of starch gelatinization and degradation. For example, lower feed rates imply a longer residence time and enhanced shearing action on starch molecules. Therefore, feed rate had a negative effect on the RDS content of extruded bean powders [8]. However, contradictory results can also be found in the literature, e.g., it was reported that the feed rate did not significantly (*p* > 0.05) affect the starch digestibility of sorghum–barley blends [98]. The contradiction of these results could be derived from the different botanical starch sources. Extruder screws can be made into different types of conveying and mixing elements (kneading and reverse screw elements). The type, length and position of the screw elements and the spacing between two elements significantly affected the molecular breakdown of starch in rice flour [113]. Different element types and screw configurations can determine the mean residence time of feeding materials by affecting the conveying efficiency, thereby changing the degree of starch degradation [113]. The shape, length and diameter of the die could affect the pressure and shear rate at the die head, thus affecting the degree of expansion and structural damage of the starch.

Overall, there are sometimes contradictory conclusions about the influence of different extrusion parameters on starch digestibility, which might be related to the botanical source of the starch. Depending on the plant source, the crystalline structure of starch and the ratio of amylose to amylopectin differ. In addition, flour is quite different from pure starch, because starch interacts with other ingredients in flour during extrusion. Therefore, from this point of view, it is impossible to draw a general conclusion about how extrusion parameters change the starch structure and digestibility, and this review can only provide partial references for explaining the mechanisms.

## 5. Conclusions and Prospects

Starch digestibility is a key factor in determining the human postprandial glycemic response, and the frequent consumption of RDS has been associated with the occurrence of many chronic diseases. Extrusion, as a common food processing technology, has a significant effect on the alteration of starch’s structure (i.e., order–disorder transition) and consequently starch digestibility. This review focused on the summarization of these effects and the possible mechanisms, with the aim to facilitate the development of starchy foods with slow starch digestibility. Extrusion could significantly degrade starch, especially amylopectin molecules around their branching points. Starch crystalline structures undergo continuous order–disorder–reorder transitions during extrusion, through starch gelatinization and retrogradation. As a result, the starch granule structure could also be largely modified. As a consequence, starch digestibility can be altered by extrusion processing. The alteration of starch digestibility by extrusion depends on the raw feed material composition, as well as the extrusion processing parameters, such as feed moisture, extrusion temperature and screw speed. This provides opportunities for developing starch-based foods with desirable starch digestibility by controlling these extrusion factors.

However, many aspects remain to be investigated in the future, in order to better apply the extrusion technique to develop starch-based foods with slow starch digestibility. For example, it is known that many factors, including the raw feed material composition, extrusion moisture, temperature and screw speed, can affect the starch structure and digestibility. However, it remains unclear how these factors combined determine starch digestibility after extrusion. Most of the past studies only focus on a single or a few of these factors. Therefore, it is still highly necessary to systematically consider the effects of all these different factors on the starch digestibility after extrusion. Furthermore, extrusion is frequently used together with other techniques, such as acid or enzymatic treatments, in order to better modify starch properties. Investigations are required to fully understand the effects of the combination of these techniques with extrusion on starch digestibility in the future. The emergence of new food extrusion techniques such as supercritical fluid extrusion technology, two-stage or multi-stage extrusion and the combination of extruders and three-dimensional (3D) printers should also be investigated in the future in order to expand the applications of extrusion in modifying starch properties.

## Figures and Tables

**Table 1:** Effects of extrusion on starch molecular structure.

Starch Type	Changes in Starch Molecular Structure	Mechanism	Reference
Genetically modified corn starches	(1) Amylopectin undergoes significant degradation during extrusion, while the size distribution of amylose does not seem to vary notably; (2) No qualitative differences in SEC weight distributions of debranched starch between native and extruded starches.	(1) The susceptibility of starch to shear degradation depends on molecular size and branching structure; (2) The shear degradation of starch during extrusion preferentially cleaves off branch points (α-1,6-linkages).	[35,44]
Rice starch	(1) Most degradation by extrusion occurs on amylopectin molecules; (2) Chain-length distribution of debranched rice starch and extruded samples reveals no significant differences.	(1) Amylopectin is more sensitive to degradation than amylose by extrusion because of its higher branching degree and larger molecular size; (2) Glycosidic bonds near amylopectin branching points are susceptible to shear degradation during extrusion.	[19,39]
Wheat starch	Amylopectin fraction with high molecular size decreases while the fraction in the molecular size range of amylose increases.	Preferential degradation on amylopectin.	[36]
Green banana flour	Amylose content increases in green banana starch after extrusion (from 16.2% to 17.3–33.5%).	Preferential cleavage of amylopectin α-1,6-glycosidic bonds under shearing force in the extruder.	[40]
Jackfruit seed starch (JFSS)	(1) Molecular weight, radius of gyration, relative crystallinity of starch are notably decreased while amylose content is not changed by improved extrusion cooking technology (IECT); (2) B2 and B3+ chains of JFSS are converted to A and B1 chains after IECT treatment.	IECT breaks α-1,4-glycosidic bonds in amylopectin backbone.	[38]
High-amylose maize flour (HAMF), normal maize flour (NMF)	Amylose content of HAMF considerably decreased, while that of NMF increased after extrusion.	The decrease in amylose content in HAMF may be induced by its high amylose and lipid content, where excessive leaching of amylose is bound with free lipids to form amylose–lipid complex during extrusion.	[41]

**Table 2:** Effects of main extrusion parameters on starch digestibility.

Extrusion Parameter	Botanic Source	Effects on Starch Digestibility	Possible Mechanisms	Reference
Feed moisture	Brown rice and pinto bean composite flours	RDS increases while RS decreases with lowered feed moisture	The lower the processing moisture, the higher the degree of mechanical degradation, resulting in a decrease in RS	[100]
	Buckwheat flour	Low moisture increases starch digestibility	Lower moisture content enhances the degree of starch molecular degradation, and thus increases starch digestibility	[97]
	Rice flour	RS increases with the increase in feed moisture	High feed moisture increases the mobility of starch molecules, leading to gelatinization, and enhances the tendency of starch to retrograde, which leads to the formation of resistant starch	[102,103]
	Corn and potato starches	High moisture decreases starch digestibility	Water has a lubricating effect inside the extruder that reduces the starch degradation. Water decreases the feed material temperature during extrusion, resulting in less gelatinization and slower starch digestion rate	[104]
Extrusion temperature	Corn starch–guar gum mixtures/Corn and potato starches	Starch digestibility increases with the increased extrusion temperature	Higher temperature results in greater starch gelatinization and thus higher starch digestibility	[59,105]
	Rice flour	RS increases with increasing temperature	High temperature causes melting of crystallites and formation of reorganized crystallites that are more stable to heat	[102,103]
	Novel wholemeal wheat flours	No relationship observed between the starch digestibility and extrusion temperatures	Starch was completely gelatinized and had no retrogradation as the samples were dried immediately after extrusion	[106]
Screw speed	Whole grain oat flour	Moderate screw speed (300 rpm) led to higher SDS and less RDS	Low screw speed means long residence time. High screw speed produces high shearing on starch molecules. Therefore, both low and high screw speed can reduce SDS	[101]
	Green banana flour	Samples extruded at lower screw speeds have higher RS content	Starch degradation is relatively less at lower screw speed, and longer residence time is favorable	[40]
	Mixture: chickpea flour, maize flour, oat flour, corn starch, onion powder	As the screw speed increases, RS decreases	Shear effect at higher screw speeds makes starch molecules more sensitive to enzymes	[107]

## Data Availability

No new data were created or analyzed in this study. Data sharing is not applicable to this article.

## References

[1] Englyst H.N., Kingman S.M., Cummings J.H. (1992). Classification and measurement of nutritionally important starch fractions. Eur. J. Clin. Nutr..

[2] Svihus B., Hervik A.K. (2016). Digestion and metabolic fates of starch, and its relation to major nutrition-related health problems: A review. Starch-Starke.

[3] Zhang G.Y., Hamaker B.R. (2009). Slowly Digestible Starch: Concept, Mechanism, and Proposed Extended Glycemic Index. Crit. Rev. Food. Sci..

[4] Keenan M.J., Zhou J., Hegsted M., Pelkman C., Durham H.A., Coulon D.B., Martin R.J. (2015). Role of Resistant Starch in Improving Gut Health, Adiposity, and Insulin Resistance. Adv. Nutr..

[5] Alam M.S., Kaur J., Khaira H., Gupta K. (2016). Extrusion and extruded products: Changes in quality attributes as affected by extrusion process parameters: A review. Crit. Rev. Food Sci..

[6] Zhang G., Ao Z., Hamaker B.R. (2006). Slow digestion property of native cereal starches. Biomacromolecules.

[7] Ottenhof M.-A., Hill S.E., Farhat I.A. (2005). Comparative study of the retrogradation of intermediate water content waxy maize, wheat, and potato starches. J. Agric. Food Chem..

[8] Cappa C., Masseroni L., Ng P.K., Alamprese C. (2020). Effect of extrusion conditions on the physical and chemical properties of bean powders. J. Food Process. Preserv..

[9] Martinez M.M., Rosell C.M., Gomez M. (2014). Modification of wheat flour functionality and digestibility through different extrusion conditions. J. Food Eng..

[10] Harper J.M., Clark J.P. (1979). Food extrusion. Crit. Rev. Food Sci. Nutr..

[11] Wei Y., Du S., Zhao X. (2009). Food Extrusion Theory and Technology.

[12] Dalbhagat C.G., Mahato D.K., Mishra H.N. (2019). Effect of extrusion processing on physicochemical, functional and nutritional characteristics of rice and rice-based products: A review. Trends Food Sci. Technol..

[13] Wang Y., Chen L., Yang T.Y., Ma Y., McClements D.J., Ren F., Tian Y.Q., Jin Z.Y. (2021). A review of structural transformations and properties changes in starch during thermal processing of foods. Food Hydrocoll..

[14] Camire M.E., Camire A., Krumhar K. (1990). Chemical and nutritional changes in foods during extrusion. Crit. Rev. Food Sci. Nutr..

[15] Cotacallapa-Sucapuca M., Vega E.N., Maieves H.A., Berrios J.D., Morales P., Fernandez-Ruiz V., Camara M. (2021). Extrusion Process as an Alternative to Improve Pulses Products Consumption. A Review. Foods.

[16] Kamau E.H., Nkhata S.G., Ayua E.O. (2020). Extrusion and nixtamalization conditions influence the magnitude of change in the nutrients and bioactive components of cereals and legumes. Food Sci. Nutr..

[17] Šárka E., Sluková M., Henke S. (2021). Changes in Phenolics during Cooking Extrusion: A Review. Foods.

[18] Patil S.S., Kaur C. (2018). Current trends in extrusion: Development of functional foods and novel ingredients. Food Sci. Technol. Res..

[19] Liu Y., Chen J., Luo S., Li C., Ye J., Liu C., Gilbert R. (2017). Physicochemical and structural properties of pregelatinized starch prepared by improved extrusion cooking technology. Carbohydr. Polym..

[20] Hayes A.M.R., Okoniewska M., Martinez M.M., Zhao B., Hamaker B.R. (2020). Investigating the potential of slow-retrograding starches to reduce staling in soft savory bread and sweet cake model systems. Food Res. Int..

[21] Li C., Wu A., Yu W., Hu Y., Li E., Zhang C., Liu Q. (2020). Parameterizing starch chain-length distributions for structure-property relations. Carbohydr. Polym..

[22] Bertoft E., Koch K., Aman P. (2012). Building block organisation of clusters in amylopectin from different structural types. Int. J. Biol. Macromol..

[23] Li C., Gong B., Hu Y., Liu X., Guan X., Zhang B. (2020). Combined crystalline, lamellar and granular structural insights into in vitro digestion rate of native starches. Food Hydrocoll..

[24] Wang S., Copeland L. (2013). Molecular disassembly of starch granules during gelatinization and its effect on starch digestibility: A review. Food Funct..

[25] Martinez M.M., Li C., Okoniewska M., Mukherjee I., Vellucci D., Hamaker B. (2018). Slowly digestible starch in fully gelatinized material is structurally driven by molecular size and A and B1 chain lengths. Carbohydr. Polym..

[26] Hoover R., Hughes T., Chung H.J., Liu Q. (2010). Composition, molecular structure, properties, and modification of pulse starches: A review. Food Res. Int..

[27] Wang S., Li C., Copeland L., Niu Q., Wang S. (2015). Starch retrogradation: A comprehensive review. Compr. Rev. Food Sci. Food Saf..

[28] Chung H.-J., Lim H.S., Lim S.-T. (2006). Effect of partial gelatinization and retrogradation on the enzymatic digestion of waxy rice starch. J. Cereal Sci..

[29] Tian J., Ogawa Y., Shi J., Chen S., Zhang H., Liu D., Ye X. (2019). The microstructure of starchy food modulates its digestibility. Crit. Rev. Food Sci..

[30] Ye J., Hu X., Luo S., Liu W., Chen J., Zeng Z., Liu C. (2018). Properties of starch after extrusion: A review. Starch-Stärke.

[31] Li C., Hu Y., Huang T., Gong B., Yu W.-W. (2020). A combined action of amylose and amylopectin fine molecular structures in determining the starch pasting and retrogradation property. Int. J. Biol. Macromol..

[32] Chi C., Li X., Huang S., Chen L., Zhang Y., Li L., Miao S. (2021). Basic principles in starch multi-scale structuration to mitigate digestibility: A review. Trends Food Sci. Technol..

[33] Vilaplana F., Gilbert R.G. (2010). Characterization of branched polysaccharides using multiple-detection size separation techniques. J. Sep. Sci..

[34] Politz M.L., Timpa J.D., Wasserman B.P. (1994). Quantitative measurement of extrusion-induced starch fragmentation products in maize flour using nonaqueous automated gel-permeation chromatography. Cereal Chem..

[35] Liu W.-C., Halley P.J., Gilbert R.G. (2010). Mechanism of Degradation of Starch, a Highly Branched Polymer, during Extrusion. Macromolecules.

[36] Cai W., Diosady L., Rubin L. (1995). Degradation of wheat starch in a twin-screw extruder. J. Food Eng..

[37] Menegassi B., Pilosof A.M.R., Areas J.A.G. (2011). Comparison of properties of native and extruded amaranth (*Amaranthus cruentus* L.—BRS Alegria) flour. LWT-Food Sci. Technol..

[38] Li B., Zhang Y., Xu F., Khan M.R., Zhang Y., Huang C., Zhu K., Tan L., Chu Z., Liu A. (2021). Supramolecular structure of Artocarpus heterophyllus Lam seed starch prepared by improved extrusion cooking technology and its relationship with in vitro digestibility. Food Chem..

[39] Liu Y., Chen J., Wu J., Luo S., Chen R., Liu C.M., Gilbert R.G. (2019). Modification of retrogradation property of rice starch by improved extrusion cooking technology. Carbohydr. Polym..

[40] Sarawong C., Schoenlechner R., Sekiguchi K., Berghofer E., Ng P.K.W. (2014). Effect of extrusion cooking on the physicochemical properties, resistant starch, phenolic content and antioxidant capacities of green banana flour. Food Chem..

[41] Zhang X., Chen Y., Zhang R., Zhong Y., Luo Y., Xu S., Liu J., Xue J., Guo D. (2016). Effects of extrusion treatment on physicochemical properties and in vitro digestion of pregelatinized high amylose maize flour. J. Cereal Sci..

[42] Htoon A., Shrestha A.K., Flanagan B.M., Lopez-Rubio A., Bird A.R., Gilbert E.P., Gidley M.J. (2009). Effects of processing high amylose maize starches under controlled conditions on structural organisation and amylase digestibility. Carbohydr. Polym..

[43] Brummer T., Meuser F., van Lengerich B., Niemann C. (2002). Effect of extrusion cooking on molecular parameters of corn starch. Starch-Starke.

[44] Li M., Hasjim J., Xie F., Halley P.J., Gilbert R.G. (2014). Shear degradation of molecular, crystalline, and granular structures of starch during extrusion. Starch-Stärke.

[45] Faraj A., Vasanthan T., Hoover R. (2004). The effect of extrusion cooking on resistant starch formation in waxy and regular barley flours. Food Res. Int..

[46] Zhang G.Y., Sofyan M., Hamaker B.R. (2008). Slowly digestible state of starch: Mechanism of slow digestion property of gelatinized maize starch. J. Agric. Food. Chem..

[47] Xu H., Zhou J., Liu X., Yu J., Copeland L., Wang S. (2021). Methods for characterizing the structure of starch in relation to its applications: A comprehensive review. Crit. Rev. Food. Sci..

[48] Pérez S., Baldwin P.M., Gallant D.J. (2009). Structural features of starch granules I. Starch.

[49] Vamadevan V., Bertoft E. (2015). Structure-function relationships of starch components. Starch-Stärke.

[50] Gallant D.J., Bouchet B., Baldwin P.M. (1997). Microscopy of starch: Evidence of a new level of granule organization. Carbohydr. Polym..

[51] Taylor N.W., Zobel H.F., White M., Senti F.R. (1961). Deuterium exchange in starches and amylose. J. Phys. Chem..

[52] Sarko A., Wu H.C. (1978). The crystal structures of A-, B-and C-polymorphs of amylose and starch. Starch-Stärke.

[53] Rodriguez-Garcia M.E., Hernandez-Landaverde M.A., Delgado J.M., Ramirez-Gutierrez C.F., Ramirez-Cardona M., Millan-Malo B.M., Londono-Restrepo S.M. (2021). Crystalline structures of the main components of starch. Curr. Opin. Food Sci..

[54] Esquivel-Fajardo E.A., Martinez-Ascencio E.U., Oseguera-Toledo M.E., Londono-Restrepo S.M., Rodriguez-Garcia M.E. (2022). Influence of physicochemical changes of the avocado starch throughout its pasting profile: Combined extraction. Carbohydr. Polym..

[55] Biliaderis C.G. (2009). Structural transitions and related physical properties of starch. Starch.

[56] Bertoft E. (2017). Understanding starch structure: Recent progress. Agronomy.

[57] Sopade P.A. (2017). Cereal processing and glycaemic response. Int. J. Food Sci. Technol..

[58] Morales-Sanchez E., Cabrera-Ramirez A.H., Gaytan-Martinez M., Mendoza-Zuvillaga A.L., Velazquez G., Mendez-Montealvo M.G., Rodriguez-Garcia M.E. (2021). Heating-cooling extrusion cycles as a method to improve the physicochemical properties of extruded corn starch. Int. J. Biol. Macromol..

[59] Von Borries-Medrano E., Jaime-Fonseca M.R., Aguilar-Mendez M.A. (2016). Starch–guar gum extrudates: Microstructure, physicochemical properties and in-vitro digestion. Food Chem..

[60] Shrestha A.K., Ng C.S., Lopez-Rubio A., Blazek J., Gilbert E.P., Gidley M.J. (2010). Enzyme resistance and structural organization in extruded high amylose maize starch. Carbohydr. Polym..

[61] Junejo S.A., Flanagan B.M., Zhang B., Dhital S. (2022). Starch structure and nutritional functionality—Past revelations and future prospects. Carbohydr. Polym..

[62] Dupuis J.H., Liu Q., Yada R.Y. (2014). Methodologies for Increasing the Resistant Starch Content of Food Starches: A Review. Compr. Rev. Food Sci. Food Saf..

[63] Roman L., Gomez M., Hamaker B.R., Martinez M.M. (2019). Banana starch and molecular shear fragmentation dramatically increase structurally driven slowly digestible starch in fully gelatinized bread crumb. Food Chem..

[64] Xie F., Liu H., Chen P., Xue T., Chen L., Yu L., Corrigan P. (2006). Starch Gelatinization under Shearless and Shear Conditions. Int. J. Food Eng..

[65] Lopez-Rubio A., Htoon A., Gilbert E.P. (2007). Influence of extrusion and digestion on the nanostructure of high-amylose maize starch. Biomacromolecules.

[66] Roman L., Campanella O., Martinez M.M. (2019). Shear-induced molecular fragmentation decreases the bioaccessibility of fully gelatinized starch and its gelling capacity. Carbohydr. Polym..

[67] Lindeboom N., Chang P.R., Tyler R.T. (2004). Analytical, biochemical and physicochemical aspects of starch granule size, with emphasis on small granule starches: A review. Starch-Stärke.

[68] Jane J.-L., Kasemsuwan T., Leas S., Zobel H., Robyt J.F. (1994). Anthology of starch granule morphology by scanning electron microscopy. Starch-Stärke.

[69] Tester R.F., Karkalas J., Qi X. (2004). Starch—Composition, fine structure and architecture. J. Cereal Sci..

[70] Amagliani L., O’Regan J., Kelly A.L., O’Mahony J.A. (2016). Chemistry, structure, functionality and applications of rice starch. J. Cereal Sci..

[71] Fannon J.E., Hauber R.J., BeMILLER J.N. (1992). Surface pores of starch granules. Cereal Chem..

[72] Huber K.C., BeMiller J.N. (1997). Visualization of channels and cavities of corn and sorghum starch granules. Cereal Chem..

[73] Huber K., BeMiller J. (2000). Channels of maize and sorghum starch granules. Carbohydr. Polym..

[74] Benmoussa M., Suhendra B., Aboubacar A., Hamaker B.R. (2006). Distinctive sorghum starch granule morphologies appear to improve raw starch digestibility. Starch-Stärke.

[75] Yan H., Zhengbiao G. (2010). Morphology of modified starches prepared by different methods. Food Res. Int..

[76] Lazou A., Krokida M. (2010). Structural and textural characterization of corn–lentil extruded snacks. J. Food Eng..

[77] Alsaffar A.A. (2011). Effect of food processing on the resistant starch content of cereals and cereal products–A review. Int. J. Food Sci. Technol..

[78] Corgneau M., Gaiani C., Petit J., Nikolova Y., Banon S., Ritie-Pertusa L., Le D.T.L., Scher J. (2019). Digestibility of common native starches with reference to starch granule size, shape and surface features towards guidelines for starch-containing food products. Int. J. Food Sci. Technol..

[79] Sharma S., Singh N., Singh B. (2015). Effect of extrusion on morphology, structural, functional properties and in vitro digestibility of corn, field pea and kidney bean starches. Starch-Starke.

[80] Al-Rabadi G.J., Torley P.J., Williams B.A., Bryden W.L., Gidley M.J. (2011). Effect of extrusion temperature and pre-extrusion particle size on starch digestion kinetics in barley and sorghum grain extrudates. Anim. Feed Sci. Technol..

[81] Robin F., Heindel C., Pineau N., Srichuwong S., Lehmann U. (2016). Effect of maize type and extrusion-cooking conditions on starch digestibility profiles. Int. J. Food Sci. Technol..

[82] Colonna P., Mercier C. (1983). Macromolecular modifications of manioc starch components by extrusion-cooking with and without lipids. Carbohydr. Polym..

[83] Holm J., Björck I., Ostrowska S., Eliasson A.C., Asp N.G., Larsson K., Lundquist I. (1983). Digestibility of amylose-lipid complexes in-vitro and in-vivo. Starch-Stärke.

[84] Singh S., Gamlath S., Wakeling L. (2007). Nutritional aspects of food extrusion: A review. Int. J. Food Sci. Technol..

[85] Bhatnagar S., Hanna M.A. (1994). Amylose-lipid complex formation during single-screw extrusion of various corn starches. Cereal Chem..

[86] Wang S., Wang J., Yu J., Wang S. (2016). Effect of fatty acids on functional properties of normal wheat and waxy wheat starches: A structural basis. Food Chem..

[87] Thachil M.T., Chouksey M.K., Gudipati V. (2014). Amylose-lipid complex formation during extrusion cooking: Effect of added lipid type and amylose level on corn-based puffed snacks. Int. J. Food Sci. Technol..

[88] Parada J., Aguilera J.M., Brennan C. (2011). Effect of guar gum content on some physical and nutritional properties of extruded products. J. Food Eng..

[89] Chung H.J., Liu Q., Hoover R., Warkentin T.D., Vandenberg B. (2008). In vitro starch digestibility, expected glycemic index, and thermal and pasting properties of flours from pea, lentil and chickpea cultivars. Food Chem..

[90] Aarathi A., Urooj A., Puttaraj S. (2003). In vitro Starch Digestibility and Nutritionally Important Starch Fractions in Cereals and Their Mixtures. Starch-Stärke.

[91] Altan A., McCarthy K.L., Maskan M. (2009). Effect of Extrusion Cooking on Functional Properties and in vitro Starch Digestibility of Barley-Based Extrudates from Fruit and Vegetable By-Products. J. Food Sci..

[92] Karkle E.L., Keller L., Dogan H., Alavi S. (2012). Matrix transformation in fiber-added extruded products: Impact of different hydration regimens on texture, microstructure and digestibility. J. Food Eng..

[93] Jafari M., Koocheki A., Milani E. (2017). Effect of extrusion cooking on chemical structure, morphology, crystallinity and thermal properties of sorghum flour extrudates. J. Cereal Sci..

[94] Mahasukhonthachat K., Sopade P., Gidley M. (2010). Kinetics of starch digestion and functional properties of twin-screw extruded sorghum. J. Cereal Sci..

[95] Govindasamy S., Campanella O., Oates C. (1996). High moisture twin-screw extrusion of sago starch: 1. Influence on granule morphology and structure. Carbohydr. Polym..

[96] Waramboi J.G., Gidley M.J., Sopade P.A. (2014). Influence of extrusion on expansion, functional and digestibility properties of whole sweetpotato flour. LWT-Food Sci. Technol..

[97] Sun X., Yu C., Fu M., Wu D., Gao C., Feng X., Cheng W., Shen X., Tang X. (2019). Extruded whole buckwheat noodles: Effects of processing variables on the degree of starch gelatinization, changes of nutritional components, cooking characteristics and in vitro starch digestibility. Food Funct..

[98] Koa S.S., Jin X., Zhang J., Sopade P.A. (2017). Extrusion of a model sorghum-barley blend: Starch digestibility and associated properties. J. Cereal Sci..

[99] Kim J., Tanhehco E., Ng P. (2006). Effect of extrusion conditions on resistant starch formation from pastry wheat flour. Food Chem..

[100] Sumargo F., Gulati P., Weier S.A., Clarke J., Rose D.J. (2016). Effects of processing moisture on the physical properties and in vitro digestibility of starch and protein in extruded brown rice and pinto bean composite flours. Food Chem..

[101] Brahma S., Weier S.A., Rose D.J. (2016). Effects of selected extrusion parameters on physicochemical properties and in vitro starch digestibility and β-glucan extractability of whole grain oats. J. Cereal Sci..

[102] Gulzar B., Hussain S.Z., Naseer B., Shikari A.B., Nazir N., Gani G. (2021). Investigation of process and product parameters on physical attributes, resistant starch, and in vitro starch digestibility of modified rice flour-based extruded snacks. J. Food Process. Preserv..

[103] Gulzar B., Hussain S.Z., Naseer B., Naik H.R. (2021). Enhancement of resistant starch content in modified rice flour using extrusion technology. Cereal Chem..

[104] Ali S., Singh B., Sharma S. (2019). Impact of Feed Moisture on Microstructure, Crystallinity, Pasting, Physico-Functional Properties and In Vitro Digestibility of Twin-Screw Extruded Corn and Potato Starches. Plant Food Hum. Nutr..

[105] Ali S., Singh B., Sharma S. (2020). Effect of processing temperature on morphology, crystallinity, functional properties, and in vitro digestibility of extruded corn and potato starches. J. Food Process. Preserv..

[106] Chanvrier H., Appelqvist I.A.M., Bird A.R., Gilbertt E., Htoon A., Li Z., Lillford P.J., Lopez-Rubio A., Morell M.K., Topping D.L. (2007). Processing of novel elevated amylose wheats: Functional properties and starch digestibility of extruded products. J. Agric. Food Chem..

[107] Ainsworth P., Ibanoglu S., Plunkett A., Ibanoglu E., Stojceska V. (2007). Effect of brewers spent grain addition and screw speed on the selected physical and nutritional properties of an extruded snack. J. Food Eng..

[108] Qi M., Zhang G., Ren Z., He Z., Peng H., Zhang D., Ma C. (2021). Impact of Extrusion Temperature on In Vitro Digestibility and Pasting Properties of Pea Flour. Plant Food Hum. Nutr..

[109] Bhatnagar S., Hanna M.A. (1994). Extrusion processing conditions for amylose-lipid complexing. Starch/Staerke.

[110] Bhatnagar S. (1993). HTST Extrusion of Starch–Lipid Systems.

[111] Kantrong H., Charunuch C., Limsangouan N., Pengpinit W. (2018). Influence of process parameters on physical properties and specific mechanical energy of healthy mushroom-rice snacks and optimization of extrusion process parameters using response surface methodology. J. Food Sci. Technol. Mys..

[112] Song C., Mao Z., Wang S., Wu M. (2008). Effects of extruding technological parameters on in vitro digestibility of flaxseed. Trans. Chin. Soc. Agric. Mach..

[113] Gautam A., Choudhury G.S. (1999). Screw configuration effects on starch breakdown during twin-screw extrusion of rice flour. J. Food Process. Preserv..

